# Urine Steroid Metabolomics as a Novel Tool for Detection of Recurrent Adrenocortical Carcinoma

**DOI:** 10.1210/clinem/dgz141

**Published:** 2019-10-29

**Authors:** Vasileios Chortis, Irina Bancos, Thomas Nijman, Lorna C Gilligan, Angela E Taylor, Cristina L Ronchi, Michael W O’Reilly, Jochen Schreiner, Miriam Asia, Anna Riester, Paola Perotti, Rosella Libé, Marcus Quinkler, Letizia Canu, Isabel Paiva, Maria J Bugalho, Darko Kastelan, M Conall Dennedy, Mark Sherlock, Urszula Ambroziak, Dimitra Vassiliadi, Jerome Bertherat, Felix Beuschlein, Martin Fassnacht, Jonathan J Deeks, Michael Biehl, Wiebke Arlt

**Affiliations:** 1 Institute of Metabolism and Systems Research, University of Birmingham, Birmingham, UK; 2 Centre for Endocrinology, Diabetes and Metabolism, Birmingham Health Partners, Birmingham, UK; 3 Department of Endocrinology, Queen Elizabeth Hospital, University Hospitals Birmingham NHS Foundation Trust, Birmingham, UK; 4 Division of Endocrinology, Diabetes, Metabolism and Nutrition, Mayo Clinic, Rochester, MN; 5 Bernoulli Institute for Mathematics, Computer Science and Artificial Intelligence, University of Groningen, Groningen, The Netherlands; 6 Division of Endocrinology and Diabetes, Department of Internal Medicine I, University Hospital, University of Würzburg, Würzburg, Germany; 7 Medizinische Klinik and Poliklinik IV, Ludwig-Maximilians-Universität München, Munich, Germany; 8 Department of Clinical and Biological Sciences, San Luigi Hospital, University of Turin, Turin, Italy; 9 INCa-COMETE, Cochin Hospital, Institut Cochin, Institut National de la Santé et de la Recherche Medicale Unite ´ 1016, René Descartes University, Paris; 10 Endocrinology in Charlottenburg, University of Berlin, Berlin, Germany; 11 Department of Experimental and Clinical Biomedical Sciences, University of Florence, Florence, Italy; 12 Department of Endocrinology, University Hospital of Coimbra, Coimbra, Portugal; 13 Serviço de Endocrinologia Diabetes e Metabolismo, Hospital de Santa Maria, Lisbon, Portugal; 14 Department of Endocrinology, University Hospital Centre Zagreb, Zagreb, Croatia; 15 School of Medicine, National University of Ireland Galway (NUIG), Galway, Republic of Ireland; 16 Department of Endocrinology, Beaumont Hospital, Dublin and the Royal College of Surgeons, Republic of Ireland; 17 Department of Internal Medicine and Endocrinology, Medical University of Warsaw, Warsaw, Poland; 18 Department of Endocrinology, Diabetes and Metabolism, Evangelismos Hospital, Athens, Greece; 19 Klinik für Endokrinologie, Diabetologie und Klinische Ernährung, Universitäts-Spital Zürich, Zürich, Switzerland; 20 Comprehensive Cancer Center Mainfranken, University of Würzburg, Würzburg, Germany; 21 Central Laboratory, University Hospital of Würzburg, Würzburg, Germany; 22 Institute of Applied Health Research, University of Birmingham, Birmingham, UK; 23 NIHR Birmingham Biomedical Research Centre, University Hospital Birmingham NHS Trust and University of Birmingham, Birmingham, UK

**Keywords:** adrenocortical carcinoma, ACC, steroid metabolomics, mass spectrometry, machine learning, recurrence detection

## Abstract

**Context:**

Urine steroid metabolomics, combining mass spectrometry-based steroid profiling and machine learning, has been described as a novel diagnostic tool for detection of adrenocortical carcinoma (ACC).

**Objective, Design, Setting:**

This proof-of-concept study evaluated the performance of urine steroid metabolomics as a tool for postoperative recurrence detection after microscopically complete (R0) resection of ACC.

**Patients and Methods:**

135 patients from 14 clinical centers provided postoperative urine samples, which were analyzed by gas chromatography–mass spectrometry. We assessed the utility of these urine steroid profiles in detecting ACC recurrence, either when interpreted by expert clinicians or when analyzed by random forest, a machine learning-based classifier. Radiological recurrence detection served as the reference standard.

**Results:**

Imaging detected recurrent disease in 42 of 135 patients; 32 had provided pre- and post-recurrence urine samples. 39 patients remained disease-free for ≥3 years. The urine “steroid fingerprint” at recurrence resembled that observed before R0 resection in the majority of cases. Review of longitudinally collected urine steroid profiles by 3 blinded experts detected recurrence by the time of radiological diagnosis in 50% to 72% of cases, improving to 69% to 92%, if a preoperative urine steroid result was available. Recurrence detection by steroid profiling preceded detection by imaging by more than 2 months in 22% to 39% of patients. Specificities varied considerably, ranging from 61% to 97%. The computational classifier detected ACC recurrence with superior accuracy (sensitivity = specificity = 81%).

**Conclusion:**

Urine steroid metabolomics is a promising tool for postoperative recurrence detection in ACC; availability of a preoperative urine considerably improves the ability to detect ACC recurrence.

Adrenocortical carcinoma (ACC) is a rare and aggressive malignancy (1, 2). Disease recurrence rates are high, even in patients with microscopically complete (R0) resection (3, 4). Therefore, vigilant surveillance of all operated patients by regular cross-sectional imaging for several years is essential to facilitate early intervention in case of recurrence (5–7). Although the optimal surveillance protocol has yet to be established, a common approach involves 3-month CT scans (thorax, abdomen, pelvis) in the first 2 postoperative years, 6-month CT scans in the next 3 years and, thereafter, annual scans until 10 years postoperatively (8). This is associated with considerable costs, repeated radiation exposure, and frequent diagnostic ambiguity in early stages of recurrent/metastatic disease (9). Early detection of disease recurrence is important, as it may allow radical revision surgery in cases of limited metastatic disease volume or timely initiation of cytotoxic chemotherapy, potentially improving survival (5, 7, 10–13). The number of metastatic sites at diagnosis of recurrent disease and time from surgery to detection of recurrence have been shown to be independent prognostic factors (10, 14).

Most ACCs are biochemically active, typically presenting a steroidogenic pattern dominated by steroid precursor metabolites rather than end products of steroidogenesis (15). This pattern has been attributed to the relative dedifferentiation of malignant cells (15, 16). Most of these steroid precursors, which represent intermediate steps along the 3 major adrenocortical steroid biosynthetic pathways, are not measured by routine clinical biochemistry. Analysis of 24-hour urine collections by gas chromatography–mass spectrometry (GC-MS), however, can identify and quantify the metabolites of the large majority of adrenal-derived steroids, providing a truly comprehensive steroid profiling tool (15). This allows the detection of minute changes in steroidogenesis and the illumination of all intermediate steps that tend to be perturbed in the setting of adrenocortical malignancy. Recent retrospective studies revealed the capacity of urinary steroid profiling to distinguish ACC from benign adrenal tumors. In 2011, our group analyzed steroid metabolite profiles in 24-hour collections from 102 patients with benign adrenocortical adenomas and 56 patients with ACC by GC-MS (15). Machine learning–based analysis of the steroid data identified a distinct malignant steroid “fingerprint” for ACC and could differentiate benign from malignant adrenal tumors with a sensitivity and specificity of 90% (15). Using GC-MS, 95% of ACCs showed evidence of steroid excess, while routine biochemistry only indicated steroid excess in 73% (15). Two subsequent retrospective studies also employing GC-MS produced similar results, albeit in smaller cohorts and without the use of machine learning analysis (16, 17).

In this study, we evaluated the diagnostic performance of urine steroid metabolomics, the combination of mass spectrometry-based steroid profiling and data analysis by machine learning-based algorithms, in the postoperative surveillance of ACC patients following microscopically complete (R0) tumor resection. We assessed the performance of this approach in the detection of disease recurrence, also comparing direct interpretation of steroid profiles by clinical experts to fully automated, machine learning–based analysis of the steroid metabolome.

## Methods

### Patients and clinical protocol

Serial postoperative 24-hour urine samples were collected from patients with histologically confirmed ACC, who had undergone microscopically complete (R0) tumor resection in 14 clinical specialist referral centers participating in the European Network for the Study of Adrenal Tumors (ENS@T; www.ensat.org), with approval of local ethical review boards and after obtaining written informed patient consent. Participating countries included the United Kingdom (Birmingham), Germany (Würzburg, Munich, Berlin), France (Paris), Italy (Florence, Turin), Greece (Athens), the Republic of Ireland (Dublin, Galway), Poland (Warsaw), Croatia (Zagreb), and Portugal (Coimbra, Lisbon). Urine samples were collected between 2007 and 2016. Inclusion criteria were defined as (i) histologically confirmed diagnosis of ACC, (ii) complete (R0) tumor resection, and (iii) provision of at least 1 postoperative 24-hour urine sample when disease-free, that is, before any radiological evidence of disease recurrence (as assessed by computed tomography [CT] thorax, abdomen, and pelvis) and within 2 years from surgery. Participating centers were prompted to provide urine samples every 3 months, but actual frequency of provided samples did not constitute an exclusion criterion as long as at least 1 postoperative sample had been provided at a time with no evidence of disease recurrence on surveillance imaging.

ACC recurrence had to be confirmed by 1 of the following: (i) emergence of new lesions on cross-sectional imaging (CT, magnetic resonance imaging), which either enlarge on follow-up scans or regress in response to systemic antitumor therapy; (ii) emergence of enhancing lesions on positron emission tomography or positron emission tomography CT scans; or (iii) histological evidence of recurrent/metastatic ACC from percutaneous biopsy or revision surgery.

### Biochemical analysis

Measurement of 24-hour urinary steroid metabolite excretion was carried out by GC-MS, as described in detail previously (15). In brief, free and conjugated steroids were extracted from 1 mL urine by solid-phase extraction. Steroid conjugates were enzymatically hydrolyzed, re-extracted, and chemically derivatized to form methyloxime trimethyl silyl ethers. GC-MS was carried out on an Agilent 5975 instrument operating in selected-ion-monitoring mode to achieve sensitive and specific detection and quantification of 19 selected steroid metabolites (Table 1) comprising 8 of the 9 previously described “malignant steroid fingerprint” metabolites indicative of ACC (15). We did not include glucocorticoid metabolites as these are uninterpretable in mitotane-treated ACC patients, who all receive high-dose glucocorticoid replacement while also being subject to the strong induction of the cortisol-metabolizing enzyme CYP3A4 by mitotane (18).

**Table 1.** Urinary steroid metabolites quantified by gas chromatography–mass spectrometry and their corresponding steroids of origin

No	Steroid Metabolite	Metabolite of
**Androgen and androgen precursor metabolites**		
1	Androsterone (An)	Androstenedione, testosterone, 5α-dihydrotestosterone
2	Etiocholanolone (Et)^a^	Androstenedione, testosterone
3	11β-hydroxyandrosterone (11β-OHAn)	Androstenedione, 11β-hydroxyandrostenedione
4	Dehydroepiandrosterone (DHEA)	DHEA, DHEAS
5	16α-hydroxy-DHEA (16α-DHEA)	DHEA, DHEAS
6	5-pregnenetriol (5-PT)^a^	17-hydroxypregnenolone
7	5-pregnenediol (5-PD)^a^	Pregnenolone
**Mineralocorticoid and mineralocorticoid precursor metabolites**		
8	Tetrahydro-11-dehydrocorticosterone (THA)	Corticosterone, 11-dehydrocorticosterone
9	5α-tetrahydro-11-dehydrocorticosterone (5α-ΤΗΑ)^a^	Corticosterone, 11-dehydrocorticosterone
10	Tetraydrocorticosterone (THB)	Corticosterone
11	5α-tetrahydrocorticosterone (5α-THB)	Corticosterone
12	3α,5β-tetrahydroaldosterone (THALDO)	Aldosterone
13	Tetrahydrodeoxycorticosterone (THDOC)^a^	11-deoxycorticosterone
**Glucocorticoid precursor metabolites**		
14	Pregnanediol (PD)^a^	Progesterone
15	3α,5α-17-hydroxypregnanolone (3α,5α-17HP)	17-hydroxyprogesterone
16	17-hydroxypregnanolone (17HP)	17-hydroxyprogesterone
17	Pregnanetriol (PT)^a^	17-hydroxyprogesterone
18	Pregnanetriolone (PTONE)	21-deoxycortisol
19	Tetrahydro-11-deoxycortisol (THS)^a^	11-deoxycortisol

These 19 steroids include 8 steroid metabolites previously described as components of the “malignant steroid fingerprint” diagnostic for adrenocortical carcinoma upon analysis of 24-hour urines from patients with benign and malignant adrenocortical masses (15).

^a^“Malignant” steroid metabolite.

### Clinical expert review of steroid profiles

Three clinical experts with extensive experience in adrenal disease (I.B., M.O.R., W.A.) were provided with longitudinally collected postoperative urinary steroid profiles from patients who either (i) developed disease recurrence (“recurrence cohort”) or (ii) remained recurrence-free over a follow-up period of at least 3 years, which we considered our “recurrence negative” cohort, as the chances of ACC recurrence past this time-point are low (19). Provision of at least 1 sample at a “disease-free” state was an essential inclusion criterion for this study; therefore, all included recurred patients had provided at least 2 postoperative urine samples (one pre- and one post-recurrence). Similarly, we only included patients from the “recurrence-free” cohort who had provided at least 2 postoperative urine samples for this study part. Preoperative steroid profiles were provided when available.

The 3 assessors were blinded to clinical and radiological information other than basic patient demographics (age, sex) and were only provided with a previously established steroid metabolite reference range derived from a healthy adult control cohort (age range 20–81 years; 77 women, 54 men). The clinical experts were asked to identify the first urine indicative of a recurrence (or state “no recurrence” in patients that they considered as non-recurred), taking into account differences of the steroid profiles to those observed in healthy controls and the previously observed “malignant steroid fingerprint” in patients with a primary ACC tumor in situ (15).

Recurrence detection by the clinical experts was considered successful only if based on interpretation of the steroid profile in a urine sample collected before or at the time of the first radiological detection of recurrent disease. This means that late biochemical detection in relation to imaging did not count as true positive for the purposes of sensitivity calculations.

### Machine learning–based data analysis

Supervised machine learning was used to create an approach for automatic separation of recurrent from non-recurrent patients (20). The machine learning algorithm was developed by presenting the results of the 19 steroid markers measured by GC-MS in a given 24-hour urine, and the corresponding output, that is, a “yes” or “no” answer to the question of whether an ACC recurrence had been radiologically detected at the time of urine collection. From these “training” examples, the algorithm learned to generalize by finding patterns in the steroid data and use them to provide an output answer when the output is not known.

We used machine learning to approach 2 separate 2-class classification problems. First, we considered the differentiation of all 215 urine samples collected in the 39 non-recurred patients from all 76 urine samples collected post-recurrence in the 32 recurred patients. Second, to test the ability of our approach for very early detection, we aimed to differentiate all non-recurred samples against the first urine sample collected in each recurrent patient at the time of first radiological detection of ACC recurrence (35 samples, as 3 of 32 patients had 2 recurrences).

Random forests were used as machine learning classifier (21, 22). The random forest is a classification framework based on the concept of decision trees. It builds a forest of many decision trees to create a strong classifier that is resistant to noise and overtraining. Another favorable property of random forests is that they give insight into the importance of features, which we exploited to inspect the contribution and relevance of each steroid metabolite in the classification problem. For all experiments, training the random forest prediction models and validating them, we used Matlab 2015a, specifically, the TreeBagger class of Matlab (included in the Statistics and Machine Learning Toolbox) (Matlab documentation, 2018). To estimate predictor importance, the parameter that controls computation of predictor importance was set to “on” (this parameter is called “oobvarimp” in Matlab 2015a). The number of decision trees used to obtain the results was 128, which provided optimal trade-off between speed and performance. Tenfold cross-validation was used to estimate the classifier’s predictive quality. To account for the differences in the number of samples between the healthy and the recurrence classes, the validation procedure was repeated 50 times for randomized splits of the data. In each run, the healthy class was randomly subsampled to make sure that both classes had an equal number of samples.

### Statistical analysis

Data analysis and graphic representation was completed using GraphPad Prism Software Version 8. Data are summarized as median (interquartile range) values unless otherwise stated. Sensitivities and specificities are accompanied by 95% confidence intervals (95% CI), derived using the Wilson/Brown method (23, 24).

## Results

### Patient characteristics

**Figure 1.**
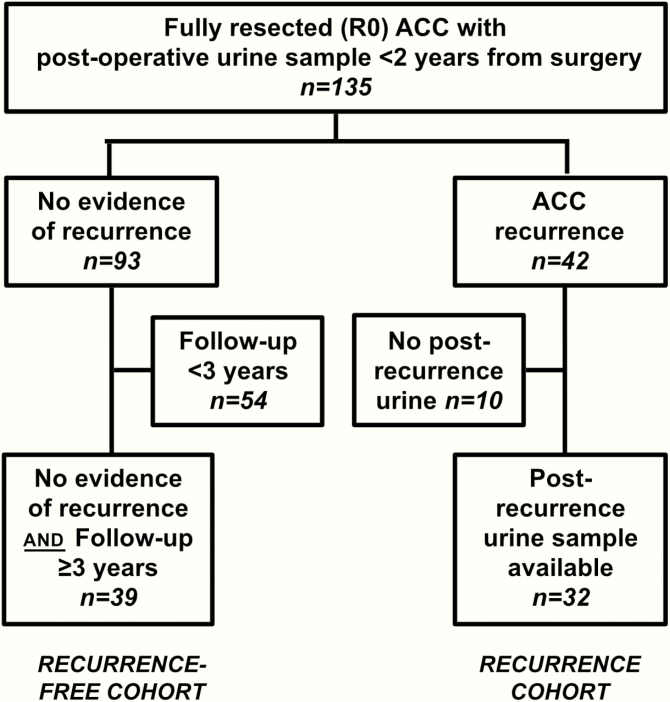
Study recruitment flow chart.

We recruited 135 patients (50 men, 85 women) who had undergone complete (R0) resection of a histologically confirmed ACC and provided at least one 24-hour urine sample while considered disease-free according to their most recent clinical and radiological assessment and no later than 2 years postoperatively (Fig. 1). Median age at diagnosis was 49 years (range 18–80 years).

During a median follow-up period of 32 months (interquartile range 15–48 months), 42 of 135 patients (31%) developed disease recurrence; of these, 10 had to be excluded from the analysis as they had not provided 24-hour urines after the detection of recurrence. Of the cohort of patients who remained disease-free postoperatively, 39 were clinically and radiologically followed for more than 3 years; as ACC recurrence presenting beyond that time frame is rare (19), we defined those 39 patients as the “recurrence-free” cohort for the purposes of this study. Relevant clinical details of both cohorts are summarized in Table 2. The remaining 54 patients without radiological evidence of recurrence but postoperative follow-up <3 years were excluded from further analysis, as they were still at high risk of potentially harboring minimal recurrent disease which had yet to manifest radiologically.

**Table 2.** Demographic and clinical characteristics

	Recurred patients (*n* = 32)	Recurrence-free patients (*n* = 39)
Postoperative 24-hour urine samples collected (total *n*)	237	216
Age (years) Median (range)	52 (22–80)	56 (24–75)
Male patients *n* (%)	7 (22%)	17 (44%)
Primary tumor size (mm) median (IQR)	92 (70–118)	80 (66–140)
Evidence of biochemical hormone excess on routine biochemistry N/total N (%)	23/31 (74%)	16/33 (48%)
Type of hormone excess on routine biochemistry (*n*)	Glucocorticoids (GC) only: 6 Androgens only: 2 Mineralocorticoids (MC) only: 0 Precursor steroids only: 0 GC + Androgens: 12 MC + GC or Androgens: 3	GC only: 5 Androgens only: 3 MC only: 1 Precursor steroids only: 1 GC +Androgens: 4 MC + GCs or Androgens: 2
Clinically overt Cushing’s syndrome *n*/total *N* (%)	16/31 (52%)	5/38 (13%)
Histology primary tumor: Ki67 (%) median (IQR)	10 (7–26)	8 (5–15)
Histology primary tumor: Weiss score (0–9) median (IQR)	5 (4–7)	5 (3–7)
Adjuvant mitotane treatment: *n* (%)	20 (63%)	27 (69%)
Duration of follow up (months) median (IQR)	27 (18–44)	51 (41–65)
Time to recurrence, (months) median (IQR)	15 (10–25)	N/A
Maximum recurrent lesion size (mm) median (range)	11 (3–45)	N/A
Number of organs involved in recurrence	1 (*n *= 27); 2 (*n* = 6); 3 (*n* = 3)	N/A
Location of recurrences	Lung (*n* = 22); Liver (*n* = 10); Lymph nodes (*n* = 5); Local recurrence (*n* = 4); Bone, spleen, omentum, pleura (each *n* = 1)	N/A

Demographics and clinical characteristics of the “recurrence” cohort of patients with disease recurrence and at least one post-recurrence urine (*n* = 32) and the “recurrence-free” cohort (patients disease-free after ≥3 years of follow-up; *n* = 39). Where data are not available for the full cohort, number of patients with available data is provided as denominator.

Abbreviations: IQR, interquartile range; N, number; N/A, not applicable.

**Figure 2.**
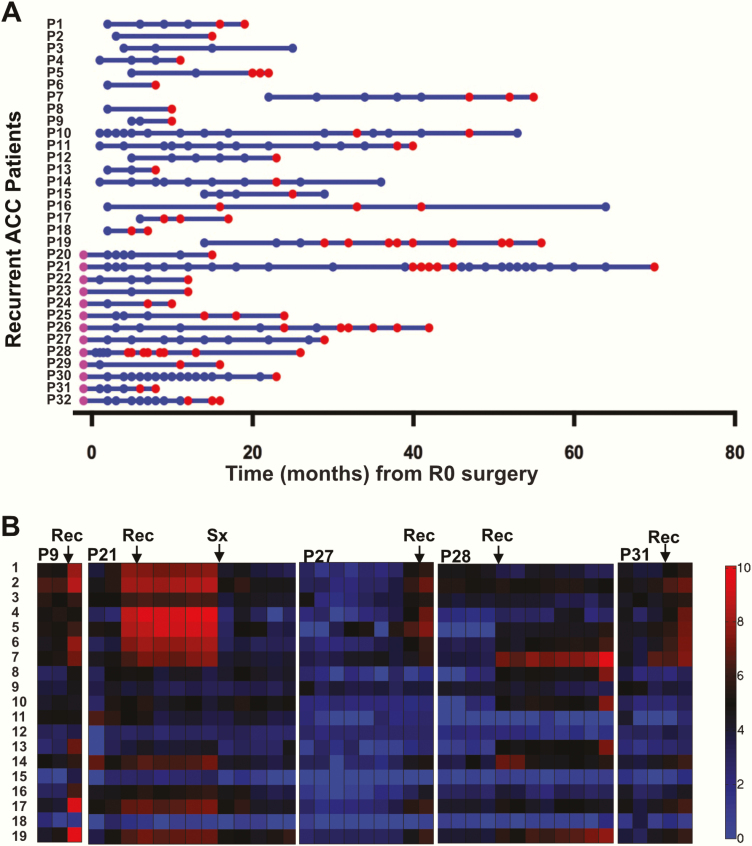
**(A)** All longitudinal urine samples collected from patients who developed disease recurrence, plotted against time from surgery. Blue dots represent postoperative samples collected while the patient was considered disease-free according to their most recent clinical and radiological evaluation. Red dots represent samples collected after the first radiological manifestation of recurrent disease and before any second curative therapy. Purple dots represent samples that were collected preoperatively. **(B)** Heat-map visualization of longitudinal urinary steroid profile results in 5 representative patients who developed recurrent disease during follow-up. Arrows indicate the time of the first radiological manifestation of recurrent disease (Rec) and surgery for recurrence (Sx). Steroid numbers correspond to steroid metabolites as tabulated in Table 1.

The 39 patients of the “recurrence-free” cohort provided a median of 4 (range 1–24) postoperative 24-hour urine samples. In the “recurrence” cohort, the patients collected a median of 5 (range 2–35) postoperative 24-hour urine samples; 13 of the 32 patients had also collected a preoperative 24-hour urine sample, facilitating the comparison of steroid profiles observed at diagnosis of the primary tumor and at detection of ACC recurrence. All samples provided by the recurred patients are depicted in Fig. 2A, plotted against time after surgery. Single-organ involvement at recurrence detection was diagnosed in 26 of the 32 recurred patients; the remaining 6 had disease affecting more than 1 organ (Table 2). We classified 15 of the 32 recurrences as “high volume” at the time of the first abnormal imaging, defined as at least 1 solid-organ lesion ≥1 cm, and 12 as “low volume”; 5 were indeterminate due to incomplete imaging information.

### Longitudinal urine steroid profiling

We hypothesized that the development of radiologically detectable recurrent or metastatic disease would be heralded by an increase in one or more adrenal steroid metabolites excreted in 24-hour urine. Such changes were indeed observed; indicative example cases are shown in heat-map format in Fig. 2B.

**Figure 3.**
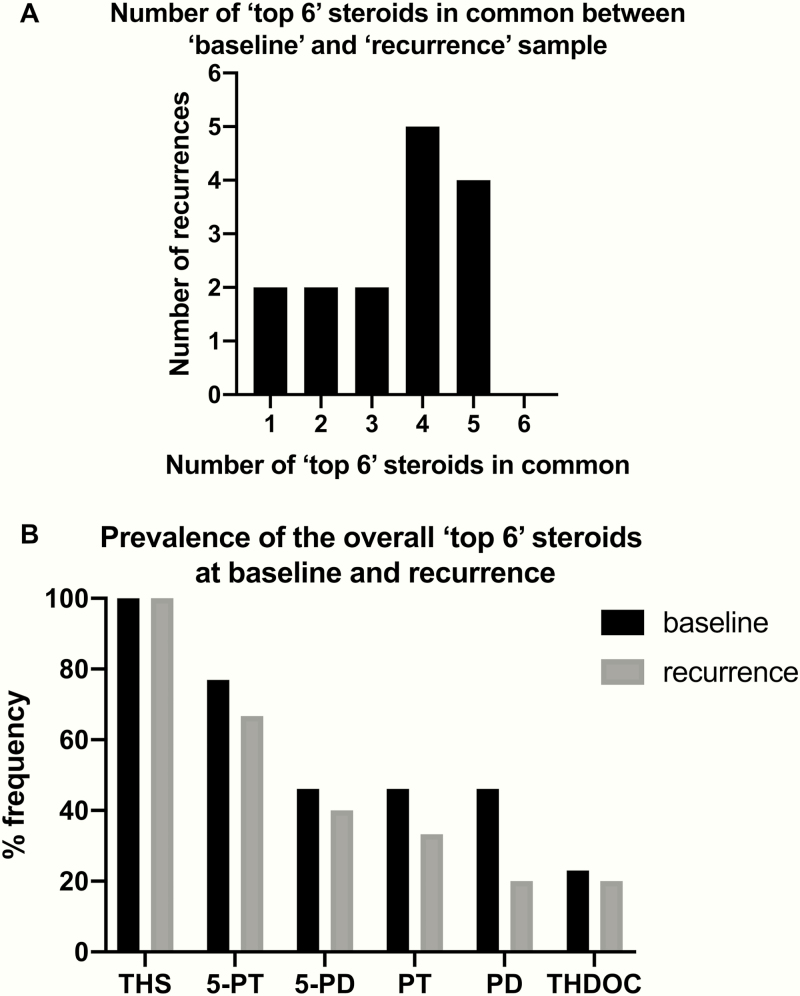
Comparison of preoperative (baseline) and post-recurrence samples (13 patients with a total of 15 recurrences). **(A)** Overlap between baseline urine steroid profile and the profile of the first post-recurrence sample provided by the same patient, when considering the 6 most elevated steroid metabolites in each sample. Steroid values were normalized to the upper limit of the reference range of the corresponding steroid metabolite in sex-matched healthy controls. **(B)** Frequency of inclusion of each steroid metabolite in the “top 6” most elevated steroid biomarkers. The presented panel consists of the 8 steroid biomarkers previously described as part of the “malignant steroid fingerprint” diagnostic for adrenocortical carcinoma (15).

An important question here was whether the “malignant steroid fingerprint” observed at baseline (ie, in the preoperative urine at the time of first diagnosis of ACC) represents an inherent characteristic of the individual ACC that is largely preserved upon disease recurrence. We found that this was indeed the case, with re-emergence of steroid metabolites at recurrence mostly identical to those found increased at baseline in the vast majority of patients (Fig. 3A). The overall 6 most increased steroids comprised the 11-deoxycortisol metabolite, tetrahydro-11-deoxycortisol (THS); the 11-deoxycorticosterone metabolite, tetrahydrodeoxycorticosterone; the pregnenolone and 17-hydroxypregnenolone metabolites, 5-pregnenediol and 5-pregnenetriol; and the progesterone and 17-hydroxyprogesterone metabolites, pregnanediol and pregnanetriol (Fig. 3B). The magnitude of steroid marker elevation, however, was substantially smaller upon disease recurrence than in primary ACCs (Table 3), as expected in view of the major differences in disease volume between primary tumor and ACC recurrence (median maximum diameter 92 vs. 11 mm, respectively; Table 2).

**Table 3.** Quantitation of the increases in the eight urine steroid metabolites previously described as part of the “malignant steroid fingerprint” diagnostic for adrenocortical carcinoma

Steroids	Preoperative sample Fold ULN Median (5th–95th percentile)	1st post-recurrence sample Fold ULN Median (5th–95th percentile)
THS	**6.57** (0.83–41.2)	**1.54** (0.35–14.50)
5-PD	**3.80** (0.79–39.4)	**0.43** (0.08–4.00)
PD	**2.93** (0.51–8.80)	**0.16** (0.03–1.10)
PT	**1.70** (0.51–10.50)	**0.22** (0.08–1.00)
5-PT	**1.69** (0.53–20.00)	**0.17** (0.04–4.40)
THDOC	**1.39** (0.15–2.90)	**0.21** (0.11–1.50)
Et	**1.19** (0.12–3.30)	**0.09** (0.02–1.00)
5α-THA	**0.47** (0.10–1.00)	**0.33** (0.09–0.51)

Steroid metabolites selected with reference to (15). Expressed as fold change in comparison to the upper limit of normal (ULN) referring to a healthy adult control cohort. We compared steroid excretion in the preoperative samples collected with the primary tumor in situ to the first urine samples collected after radiological recurrence detection ( = 1st post-recurrence sample) in the 13 patients with ACC recurrence who provided both pre- and postoperative urine samples.

Abbreviations: THS, tetrahydro-11-deoxycortisol; 5-PD, 5-pregnenediol; PD, pregnanediol; PT, pregnanetriol; 5-PT, 5-pregnenetriol; THDOC, tetrahydrodeoxycorticosterone; Etio, etiocholanolone; 5α-THA, 5α-tetrahydro-11-dehydrocorticosterone.

### Expert clinician assessment of steroid profiling results

**Figure 4.**
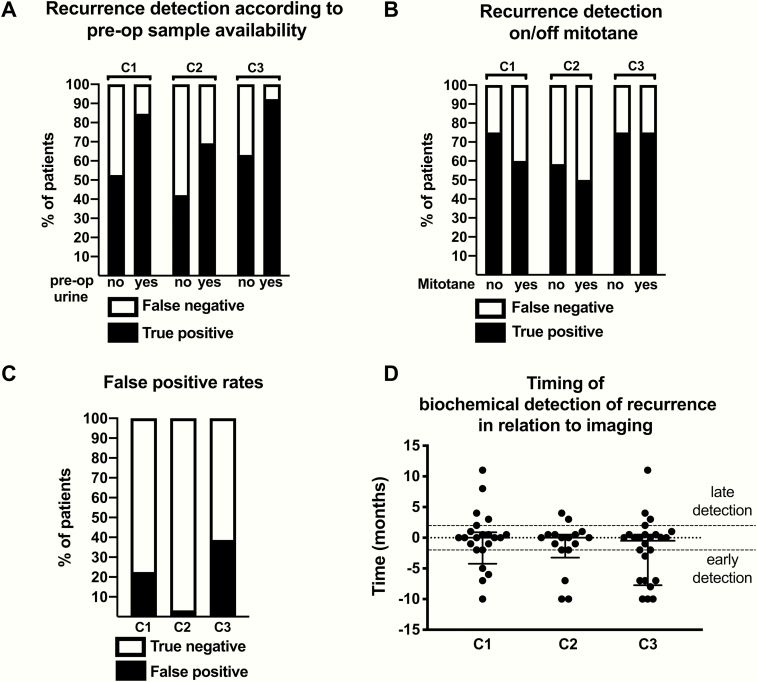
Assessment of longitudinally collected urine steroid profiling results by 3 expert clinicians (C1–C3). **(A+B**) Clinician assessment in the 32 patients who developed disease recurrence, grouped according to whether they had provided preoperative urine samples (*n* = 13) or not (*n* = 19) (**A**) or whether they had adjuvant mitotane treatment at the time of recurrence (*n* = 12) or not (*n* = 20) (**B**). **(C)** Clinician assessment of urine profiles from 31 patients who remained disease-free for at least three years post-operatively and provided at least two post-operative samples. **(D)** Time interval between the detection of recurrent adrenocortical carcinoma by imaging and the earliest detection by clinician assessment of urine steroid profiles. Each point corresponds to a single urine sample; no patient is represented by more than one sample. Negative values indicate that biochemical detection preceded radiological detection.

To evaluate the extent to which incipient steroid profile changes can facilitate diagnosis of recurrent disease, we asked 3 expert endocrinologists to retrospectively review longitudinal series of urinary steroid profiles individually derived from the “recurrence” and “recurrence-free” patient cohorts. The 3 expert clinicians were able to correctly identify recurrent disease by the time of the first post-recurrence sample (defined by reference to the first abnormal surveillance scan) with sensitivities of 66% (95% CI 48–80%), 53% (95% CI 36–69%), and 75% (95% CI 58–87%) (clinicians 1, 2, and 3, respectively). This improved substantially for the subgroup of patients who had provided preoperative urine samples (*n* = 13) to 85% (95% CI 58–97%), 69% (95% CI 42–87%) and 92% (95% CI 67–100%), respectively (Fig. 4A). Of note, 8 of the 13 assessed recurrences in these patients were unanimously detected by all 3 reviewing clinicians. Absence of a preoperative sample curtailed diagnostic sensitivities to 53% (95% CI 32–73%), 42% (95% CI 23–64%), and 63% (95% CI 41–81%), respectively.

The diagnostic performance of the steroid profile review was not altered by adjuvant mitotane treatment (Fig. 4B). Whether the tumor was found to be hormonally active or not at baseline (on clinical biochemistry) also did not appear to affect the diagnostic performance of the urine steroid metabolome on recurrence (data not shown).

The proportion of non-recurred patients in whom recurrences were incorrectly detected by reviewing clinicians varied considerably across the assessors (false positive rates 23% [95% CI 11–39%], 3% [95% CI 0–16%], and 39% [95% CI 24–56%] for clinicians 1, 2 and 3, respectively; Fig. 4C). The effect of the availability of a preoperative urine sample on the specificity of detection could not be meaningfully assessed as only 5 non-recurred patients had provided a preoperative sample.

Tumor volume at the time of first abnormal surveillance imaging was a second clinical factor, which showed a tendency toward affecting clinician ability to detect recurrence (high-volume recurrence: sensitivities 67% [95% CI 42–85%], 53% [95% CI 30–75%] and 87% [95% CI 62–98%]; low-volume recurrence: 50% [95% CI 25–75%], 42% [95% CI 19–68%] and 58% [95% CI 32–81%] for clinicians 1–3, respectively).

Of note, a considerable proportion of correct recurrence detections (ranging from 22%–39% for the 3 experts) were made based on urine collections that predated the first radiological evidence of recurrence by more than 2 months (Fig. 4D). Only a small number of recurrences were detected later by urine steroid profile interpretation than radiological detection. If late detections were accepted as positive, the overall sensitivities of the clinicians would improve to 75% (95% CI 58–87%), 56% (95% CI 39–72%), and 81% (95% CI 65–91%) for clinicians 1–3, respectively.

### Computational analysis of steroid data

**Figure 5.**
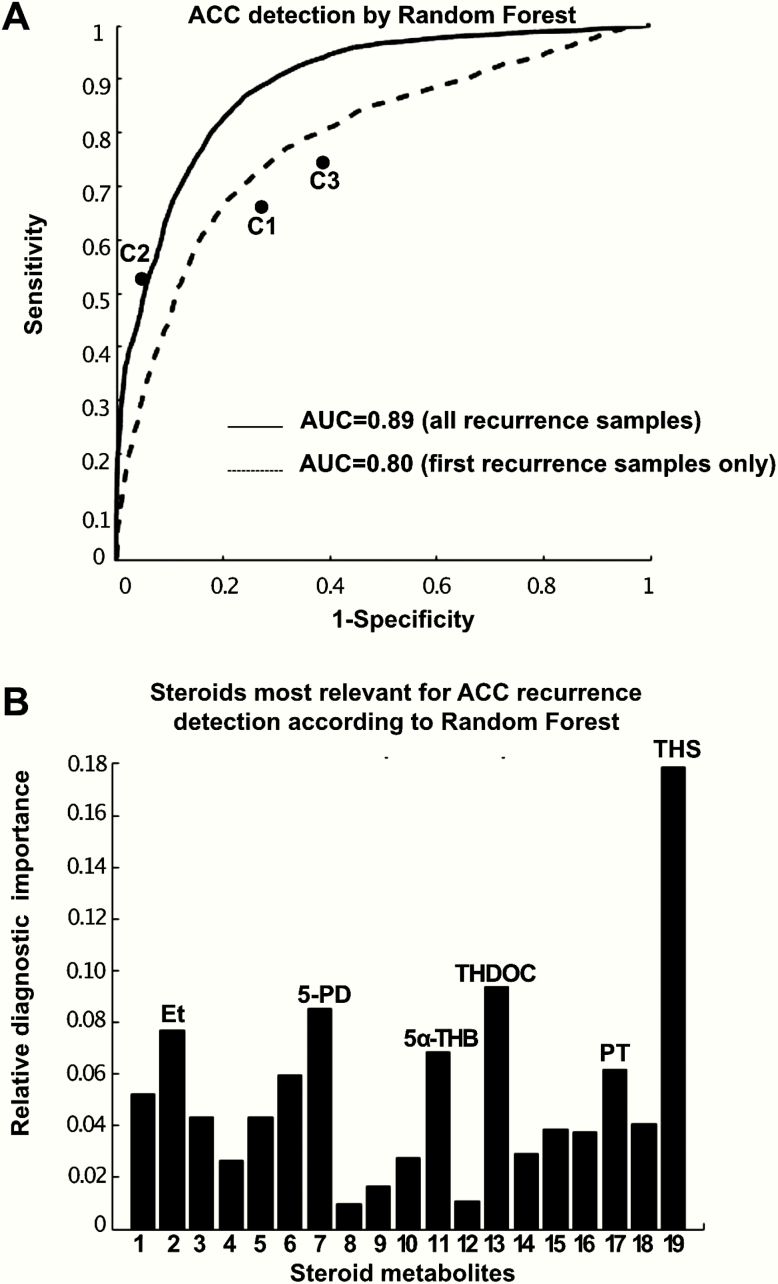
Machine learning-based analysis of the urine steroid profile results. **(A)** Receiver operating characteristics curve analysis of the performance of random forest classification in distinguishing post-recurrence samples from samples provided by non-recurred patients. The performance of the three clinician assessors (C1–C3) has also been plotted for comparison. Steroid numbers correspond to Table 1. **(B)** Random forest assessment of the relative importance of the 19 steroid metabolites in distinguishing post-recurrence samples (*n* = 32) from postoperative samples provided by non-recurred patients (*n* = 39), quantifying the significance of each single steroid marker for the detection of adrenocortical carcinoma recurrence, with all significances adding up to the sum of 1.

Machine learning-based analysis of the urine steroid profile data by random forests were able to distinguish post-recurrence urine samples (*n* = 76) provided by the 32 recurred patients from postoperative urine samples (*n* = 215) provided by the 39 non-recurred patients with high accuracy (85%; area under the receiver operating characteristic curve (AUROC) 0.89, 95% CI 0.86–0.91; sensitivity = specificity = 81%) (Fig. 5A).

When considering only the first post-recurrence urine sample from each patient in the “recurrence” cohort, the accuracy of random forest classification was lower at 75% (AUROC 0.80, 95% CI 0.75–0.85) (Fig. 5A), with the urine of 18 of 32 patients correctly classified as indicative of ACC recurrence (sensitivity 56%, CI 39–72%); in 6 of these patients, recurrence was also detected in samples that preceded the first abnormal imaging. Applying the same diagnostic cut-off, 79% of samples provided by patients in the “non-recurred” cohort were correctly identified as non-recurred (false positive rate 21% [95% CI 16%–27%]).

High-volume recurrences were more likely to be detected by the random forest classifier (sensitivity 60% [95% CI 36%–80%] vs. 42% [95% CI 19%–68%] in low-volume recurrences). However, detection was not affected by mitotane treatment (sensitivity 55% [95% CI 34%–74%] in mitotane-treated patients vs. 58% [95% CI 32%–81%] in patients not treated with mitotane). Interestingly, in 11 of 18 successfully diagnosed recurred patients, recurrence had also been unanimously detected by all 3 assessing clinicians and in 16 of 18 by at least 2 different clinicians.

The machine learning analysis determined the 11-deoxycortisol metabolite THS as the single most important steroid metabolite in differentiating post-recurrence urine samples from samples provided by non-recurred patients, followed by the mineralocorticoid precursor metabolite tetrahydrocorticosterone, the pregnenolone metabolite pregnenediol and the androgen metabolite etiocholanolone (Fig. 5B).

## Discussion

In this study, we explored the utility of urinary steroid profiling as a novel diagnostic tool for recurrence detection in patients with microscopically complete (R0) resection of ACC. Results show that analysis of the steroid profiling data by a machine learning–based algorithm represents a highly promising noninvasive and radiation-free tool. Once validated prospectively, this would be a useful addition to the current imaging-focused follow-up protocols, expediting scans in patients with suspicious biochemistry and informing discussions in cases with ambiguous imaging results.

Urinary steroid profiling in conjunction with machine learning-based data analysis, also termed urine steroid metabolomics, has already yielded highly promising results in several retrospective studies in patients with primary adrenal masses, where it was employed to differentiate ACCs from benign adrenal tumors (15, 16). In the distinct clinical setting of postoperative patient surveillance after resection of ACC, the use of urine steroid profiling has only been reported in a few cases (25, 26), but has never been systematically investigated.

In the present study, we studied 135 adult patients with microscopically complete R0 resection of ACC recruited from 14 centers associated with the European Network for the Study of Adrenal Tumors (ENSAT). Of the 81 patients who completed 3 years of postoperative surveillance, 42 (52%) recurred, a rate that is similar to previous retrospective studies (19, 27), which suggests that our patient cohort is representative of ACC patients routinely seen in clinical practice.

An important finding of our study is that there were substantial similarities between the steroid profiles of recurrent ACCs and their respective steroid profiles collected preoperatively, with the primary tumor in situ. Indeed, half of all recurrent ACCs shared 4 or 5 of their “top 6” most elevated steroid metabolites with their primary tumor of origin. Most of the “malignant steroid biomarkers” that were identified in our 2011 study on primary adrenal tumors (15) were also highly relevant in the context of recurrent disease, comprising the “top 6” increased metabolites detected in urines collected from patients with ACC recurrences. Consequently, expert clinicians had improved ability to detect recurrence if a preoperative urine steroid profile was available. This emphasizes the importance of preoperative, baseline sample collection to facilitate personalized management in patients with ACC, a rare cancer in which baseline tissue and blood collection is increasingly becoming routine to support individualized diagnosis and therapy (28).

We assessed the diagnostic potential of urinary steroid profiling as a recurrence surveillance tool using two approaches: (i) an “expert review” approach and (ii) automatic recurrence detection by computational analysis of steroid data using a machine learning–based algorithm. On retrospective, blinded assessment of serial 24-hour urine collections, clinicians were able to detect recurrence by the time of its first radiological manifestation with high sensitivity in cases where a preoperative urine sample was available. In patients who were only able to contribute postoperative urine samples, the ability of clinicians to detect recurrence was substantially lower.

Adjuvant mitotane did not compromise the diagnostic performance of reviewing clinicians, despite the drug’s well documented ability to inhibit steroidogenesis (18). Mitotane interferes with adrenal steroidogenesis in a number of ways, including (i) overall suppression of steroidogenesis resulting in lower excretion values for all steroid metabolites, (ii) increased glucocorticoid breakdown by induction of CYP3A4, necessitating high-dose hydrocortisone replacement; and (iii) 5α-reductase inhibition, leading to a decrease in 5α-reduced steroids (18). Although mitotane appeared to blunt the magnitude of the increases in ACC-specific steroid biomarkers in recurred patients, it also suppressed the random sample-to-sample variability, which can be diagnostically helpful. We systematically excluded glucocorticoid metabolites from the steroid analysis, as these would be compromised both by mitotane-induced changes in glucocorticoid metabolism and exogenous hydrocortisone replacement.

We applied a machine learning–based approach to the urinary steroid profiling data to detect recurrent ACC in an automated and defined fashion. The biochemical complexity of steroidogenesis with multiple substrates, products, and pathways, in combination with the small underlying disease volumes in the setting of recurrent malignancy, render individual biomarkers diagnostically insufficient. Machine learning–based approaches are ideally suited to systematically evaluate the wealth of data provided by multisteroid profiling in an objective and reproducible fashion, as already demonstrated in the differential diagnosis of adrenal incidentalomas (15). Our classifier could distinguish recurred samples from samples provided by non-recurred patients with considerable accuracy. THS was the most important indicator of malignancy, reflecting the pattern of inefficient steroidogenesis in ACC that emerged in previous studies on detection of ACC in patients with adrenal masses (15, 17, 29). Indeed, all but one of the 6 steroids that were identified by random forest as most differentiating between recurrence and non-recurrence are contained in the previously described “malignant steroid fingerprint” in ACC (15). It should be noted that, unlike assessing clinicians, the computational classifier did not take into account the dynamic longitudinal changes in steroid metabolites in individual patients but judged every sample on its own.

To our knowledge, this is the first study systematically exploring the diagnostic potential of urine steroid profiling in the postoperative monitoring of ACC patients. Strengths of our study include the large cohort size and the application of computational analysis to meet the demands of the multivariable GC-MS steroid datasets. The limitations of our work pertain to the paucity of preoperative samples in the majority of patients, the variable frequency of postoperative sample collections and the fact that the machine learning classifier has not been validated on an additional data set. We also did not systematically compare the results of routine biochemical analysis of serum steroids to the 24-hour urine analysis by GC-MS; however, we previously demonstrated that routine serum biochemistry only identified abnormalities in 73% of ACC patients (*n* = 47), while urine steroid metabolomics by GC-MS found abnormalities in 95% (15).

On this background, and despite the generally small disease volume in the recurred patients, in comparison to patients presenting with a large primary tumors (15), our approach yielded very promising diagnostic results. Our data indicate that availability of a preoperative urine and, thus, of the preoperative “steroid fingerprint” considerably improves the likelihood of recurrence detection and, therefore, the preservation of a preoperative 24-hour urine sample should be routinely considered, in addition to preservation of serum, plasma, and tissue, to facilitate precision medicine.

In conclusion, we demonstrated that urine steroid metabolomics, that is, the combination of mass spectrometry–based steroid profiling with machine learning–based steroid data analysis, is superior to interpretation of steroid profile results by individual experts. Following potential further refinement of this algorithm, this diagnostic approach should be taken forward to be assessed against radiological disease detection in a prospective test validation study with systematic collection of pre- and postoperative urines in defined intervals. This will also allow for systematic comparison of serum and 24-hour urine steroid profiles and ideally utilize high-throughput technology, such as liquid chromatography–tandem mass spectrometry or also, as recently published (30), high resolution accurate mass spectrometry, both assays highly suitable for rollout of urine steroid metabolomics into the routine clinical context.

## Additional Information

***Disclosure Summary:*** W.A. is an inventor and M.B. is a contributor to a patent on the use of steroid profiling as a biomarker tool in the differential diagnosis of steroid-producing and steroid-dependent tumors (PCT/GB2010/000274). I.B. reports advisory board participation with HRA Pharma outside the submitted work. All other authors did not declare a conflict of interest. The views expressed are those of the authors and not necessarily the official views of the UK National Institute for Health Research, the UK Department of Health and Social Care, or the US National Institutes of Health.

***Data Availability:*** The datasets generated during and/or analyzed during the current study are not publicly available but are available from the corresponding author on reasonable request.
